# Mutation accumulation in *H*. *sapiens* F508del *CFTR* countermands dN/dS type genomic analysis

**DOI:** 10.1371/journal.pone.0305832

**Published:** 2024-07-18

**Authors:** Jeong S. Hong, Janice M. Tindall, Samuel R. Tindall, Eric J. Sorscher

**Affiliations:** Emory University School of Medicine, Atlanta, Georgia, United States of America; University of Alabama at Birmingham, UNITED STATES

## Abstract

Understanding the mechanisms that underlie *de novo* mutations (DNMs) can be essential for interpreting human evolution, including aspects such as rapidly diverging genes, conservation of non-coding regulatory elements, and somatic DNA adaptation, among others. DNM accumulation in *Homo sapiens* is often limited to evaluation of human trios or quads across a single generation. Moreover, human SNPs in exons, pseudogenes, or other non-coding elements can be ancient and difficult to date, including polymorphisms attributable to founder effects and identity by descent. In this report, we describe multigenerational evolution of a human coding locus devoid of natural selection, and delineate patterns and principles by which DNMs have accumulated over the past few thousand years. We apply a data set comprising cystic fibrosis transmembrane conductance regulator (*CFTR*) alleles from 2,393 individuals homozygous for the F508del defect. Additional polymorphism on the F508del background diversified subsequent to a single mutational event during recent human history. Because F508del CFTR is without function, SNPs observed on this haplotype are effectively attributable to factors that govern accumulating *de novo* mutations. We show profound enhancement of transition, synonymous, and positionally repetitive polymorphisms, indicating appearance of DNMs in a manner evolutionarily designed to protect protein coding DNA against mutational attrition while promoting diversity.

## Introduction

Tabulations of non-synonymous and synonymous human single nucleotide polymorphisms (SNPs) have been applied to a large body of scientific literature as the best available means to characterize rates of gene evolution and other features of DNA diversification. Knowledge in this area is derived from a reasonable assumption that *de novo* SNPs appear in essentially random fashion, and that SNP distribution patterns revealed by sequence analysis—whether for an individual or population—are best attributed to natural selection, drift, or related processes. Such an assumption has recently been questioned by a growing number of reports, including studies from *Homo sapiens*, Arabidopsis, single cell organisms, and viral pathogens [1–9]. Measurements of DNMs in large human cohorts, for example, show variation in SNP frequency that may differ by 100-fold at certain loci—a far greater magnitude than more modest differences commonly used to infer effects of evolutionary selection. Quantitative arguments and assemblages of DNA sequence data indicate that conclusions based on non-synonymous to synonymous proportions (e.g., dN/dS) or other widely used metrics may need to be revisited [1, 2, 5, 6, 9].

Testing the extent to which SNPs originate randomly (either at a particular position or throughout human DNA) is problematic. For exonic SNPs identified by next-generation sequencing initiatives such as gnomAD, for example, whether a specific mutational scenario is evolutionarily advantageous or simply the result of mutational “hot spots” or “cold zones” usually remains unknown [9, 10]. Frequency of certain SNP categories such as pLOF (potential loss of function) variants comprise part of the raison d’être for gnomAD, based on the notion that low pLOF frequency in a particular gene should reflect purifying selection and provide a method for identifying loci that are essential (i.e., poor targets for pharmacologic intervention). However, many human genes with low pLOF counts are non-essential or expendable [9]. Moreover, determining whether human *de novo* SNP formation can skew ratios of transition, synonymous, pLOF, or other polymorphisms is complicated by extreme rarity of these events (approximately 100 *de novo* point mutations per 3 billion human nucleotide positions per generation). Although many hundreds of published reports have assumed DNMs (synonymous versus non-synonymous; transition versus transversion) are sufficiently random to apply dN/dS, pLOF frequency, or comparable statistical tools, it has been quantitatively difficult (or impossible) to formally test that assertion.

The present study applied a unique DNA database together with emerging functional genomic, biochemical, and phenotypic knowledge regarding the cystic fibrosis transmembrane conductance regulator (*CFTR*) to address a number of key evolutionary questions. In particular, the common F508del mutation abolishes CFTR function in human, murine, rat, ferret, rabbit, and porcine cells, tissues, and organisms [11–17]. The pathogenic mechanism is unambiguous and attributable to premature endoplasmic reticulum-associated degradation. As a consequence, negligible F508del CFTR arrives in a functional form at the plasma membrane, and if mutant protein is rescued to the cell surface (e.g., using small molecules that overcome the F508del maturational processing abnormality), F508del nonetheless confers severe gating and cell surface stability defects [12, 18–20]. Because F508del CFTR is without significant activity, it would be extremely unlikely for new SNPs on the mutant protein background to be the subject of further positive or negative selection. (The protein is inactive; an argument that new SNPs on an F508del allele might elicit additional fitness effects would contradict much of what has been learned regarding CFTR during the past 30 years.) Moreover, the F508del protein has been extensively evaluated for partial suppressor mutations, and although a few such SNPs are known, none are germane to analysis presented here [21, 22] (see also below).

F508del is associated with a single *CFTR* haplotype and believed to have originated only once–somewhere between ~1,100 and 52,000 years ago in a human from Northern Europe [23, 24]. Homozygosity for F508del *CFTR* causes the disease cystic fibrosis, which is fatal in childhood if untreated, and (in the absence of *in vitro* fertilization technology) precludes reproduction among males [25]. In addition, since F508del is quite recent in evolutionary terms, epistasis and small changes in fitness over a protracted time scale are less relevant. Moreover, CF is an autosomal recessive condition, and complete loss of one allele (approximately 1 in 30 Caucasians is a silent CF carrier) has minimal effect on human health (i.e., CFTR overwhelmingly exhibits haplosufficiency). A novel SNP on an F508del background, therefore, would not confer additional deleterious effects (F508del is already non-functional) and would be extremely unlikely to elicit positive selection in a CF carrier (the wt CFTR at this locus is already fully functional; i.e., there is no dominant negative effect). Studying recent SNPs on the F508del haplotype therefore provides a powerful opportunity to observe human missense mutations that have accumulated at an otherwise intact and active protein coding locus over a comparatively brief time interval and limited number of *H*. *sapiens* generations. Such an analysis minimizes confounding effects of epistasis or natural selection in a species for which cellular and tissue physiology is unlikely to have changed much since the original F508del mutation occurred.

In this report, we examined SNP formation bias and the premise that DNMs are sufficiently random to allow accurate and meaningful evolutionary conclusions to be drawn. We describe the types and locations of SNPs that have accumulated in 2,393 patients with CF homozygous for the F508del variant. Our results show strongly non-random patterns of SNP accrual that: 1) cannot be attributed to natural selection, 2) indicate pronounced bias in *de novo* SNP formation, and 3) belie the time-honored and broadly utilized assumption that SNPs are generated in a manner suitable for evolutionary inference.

## Methods

**Fig 1:**
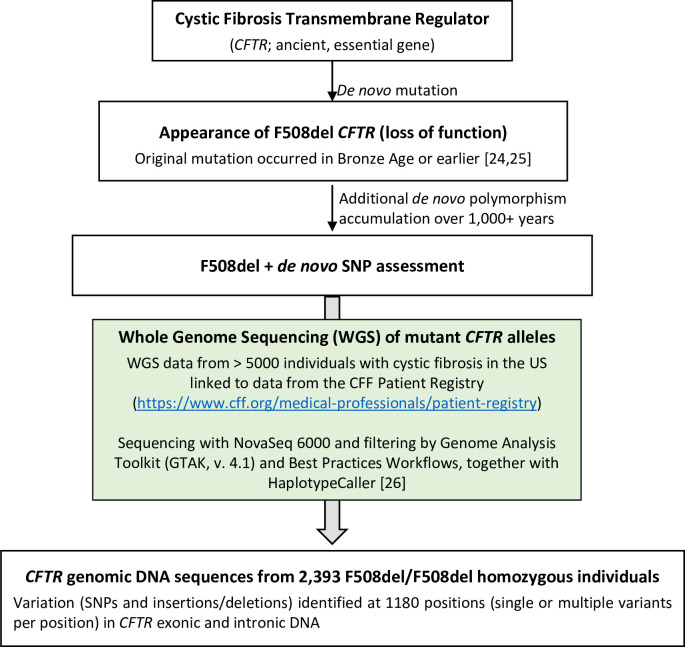
Overall study design, including methods to identify polymorphisms on an F508del *CFTR* allelic background.

Next-generation sequencing and high stringency validation of *CFTR* DNA from over 5,000 individuals with cystic fibrosis has been reported previously by Raraigh *et al*. [26]. Workflow for analysis of F508del alleles from that data repository and used by the present study is summarized in Fig 1. Sequence and SNP curation by investigators at five collaborating institutions is available to the scientific community [26]. Computer code for our project is provided under **Supporting information**, and discussed below.

### Computer modeling

A computer simulation was performed using Python and 184,202 potential DNA positions (size of *CFTR*) to model locations at which a SNP might occur. Single nucleotide changes were simulated one at a time and placed randomly in the target sequence. After 969 SNPs had been modeled for each run (see Table 1), the simulation was checked for numbers of residues receiving more than a single point mutation.

**Table 1:** Polymorphisms identified in 4,786 F508del CFTR alleles.

Exonic	Observed	Expected*	Expected**
Non-synonymous:synonymous	25:15 = 38% syn.	3.2:1 = 24% syn.	3.3:1 = 23% syn.
Transition:transversion	24:16 (includes one stop codon) = 60% transition	0.5:1 = 33% transition	0.5:1 = 33% transition
% of coding transition mutations leading to synonymous SNPs	11/24 = 46%	34%	34%
**Intronic**	Observed		Expected*
Transition:transversion	585:344 = 63% transition		0.5:1 = 33% transition
*If stochastic and based on genetic code corrected for codon usage **Computer modeling of all possible mutations at every CFTR exonic position Non-synonymous:synonymous (observed versus expected) p = 0.027 Transition:transversion (observed versus expected) p = 0.00049			
	Observed		
Non-coding versus coding mutations (from a total of 969 SNPs)	929:40 = 23 times increased non-coding compared to coding SNPs		

To assess SNP distribution ratios and types of mutations occurring in *CFTR* exons, every coding SNP generated by simulation was recorded and the amino acid produced determined. Computer-generated mutations were next categorized as to whether the modified position led to a transition or a transversion, and whether the resulting amino acid was synonymous, non-synonymous, or a nonsense variant. Mutation placement for each simulation used the human *CFTR* DNA sequence with each codon referenced against codon usage [9].

### Statistical analysis

SNPs generated by random computer simulation were distinct from those experimentally observed for F508del *CFTR* alleles. A binomial distribution calculator was used to determine probability of observed SNP ratios in comparison to computer modeling data. p-value < 0.05 was taken as significant.

## Results and discussion

**Fig 2:**
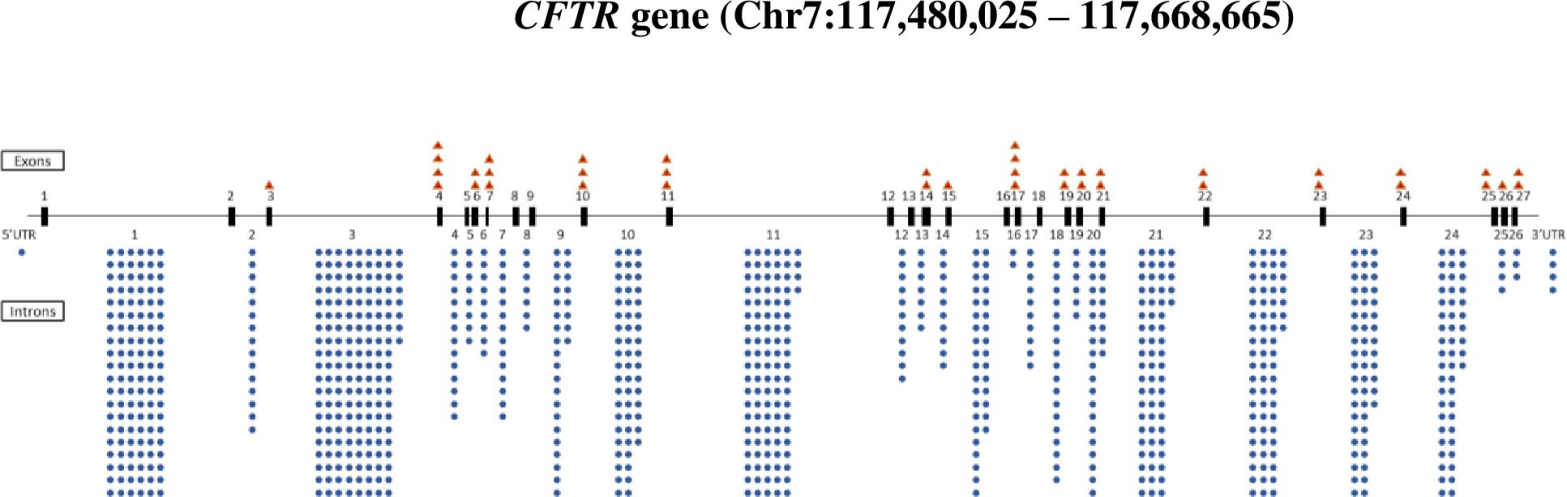
Distribution of SNPs identified in 2,393 patients with cystic fibrosis homozygous for F508del *CFTR*. SNP counts are shown as orange triangles (exonic) or blue circles (intronic and 3’ UTR).

SNP accumulation findings are described in Fig 2 and Table 1.

### Transition versus transversion polymorphisms

Transition SNPs (T↔C, G↔A) have been measured in numerous previous studies of species comparison, DNM trios, tumor genomes, etc., and are typically present at far higher numbers than would be expected by chance alone. CpG methylation can be invoked to explain such findings, but accounts for only a fraction of transitions, based on experiments in yeast (where DNA methylation is very rare) and DNM analysis in humans (where transition SNPs are strongly enhanced at both CpG and non-CpG locations) [9, 27]. On the F508del haplotype, transition SNPs have accumulated in marked preference to transversions (585 intronic transition SNPs versus 344 transversions (63% transition); 24 exonic transition SNPs versus 16 transversions (60% transition)). The expected proportion, which favors transversions by a ratio of 2 to 1, has therefore nearly been reversed (Table 1).

What is the significance of transition SNP enhancement? Among other features, transitions confer synonymous polymorphism as programmed by the genetic code [1, 28, 29]. The tendency to elicit synonymous mutations is especially pronounced for transitions at the 3^rd^ nucleotide position of a codon (the preferred site for transition polymorphisms), where 94% of transitions lead to a synonymous amino acid change. Transition preference as shown in Table 1 cannot be accounted for by purifying selection of a functionless CFTR protein (note also that strong transition preference in coding DNA is very similar to non-coding elements, further arguing against recent selective pressure as an explanation). The increase of transition SNPs on an F508del *CFTR* background also agrees with a previous study of human DNMs among 283 trios, where ~65% of DNMs were transitions [27]. Such findings strongly contradict the notion of random SNP production. DNM bias of this type would serve evolutionarily to preserve protein coding sequences by favoring synonymous SNPs (Table 1) and limit genomic attrition (so called “meltdown”) [1, 2, 9].

### Non-synonymous versus synonymous SNPs

Over the past 50 years (and continuing at present), very large numbers of published reports have drawn conclusions about human genomic evolution based on adjusted non-synonymous to synonymous SNP ratios (dN/dS) or similar quantitative methods. An unproven supposition for these studies has been that diminished numbers of non-synonymous SNPs reflect purifying selection that removes deleterious mutations from the gene pool.

Data from sequence analysis of 2,393 individuals with CF who are homozygous for F508del *CFTR* establishes a synonymous SNP incidence of ~38%. This result has meaning, since the percentage of synonymous DNMs genome-wide in human DNA has been reported previously to be of the same magnitude (approximately 30–44%) among normal controls and patients with autism [30–32]. Our findings therefore indicate that in contrast to many traditional applications, the primary determinant for non-synonymous to synonymous SNP ratios may simply reflect *de novo* mutation bias (as opposed to purifying selection or classic constraint). We note that in semblance to human DNM data (indicating up to 100-fold differences in mutational frequency at specific loci genome-wide), our findings further contradict use of non-synonymous SNP depletion as a routine means for evaluating DNA evolution. Note that for human genes reportedly subject to either rapid evolution or strong negative selection, values of non-synonymous SNPs may only be a few percentage points from “expected”. Data from gnomAD, for example, indicates typical synonymous SNP percentages of 29–40% across many loci (including both essential and non-essential genes)—a value used to indicate magnitude of constraint but very similar to the F508del synonymous proportion shown here—despite clear evidence that DNM formation bias (and not purifying selection) is responsible in the present study.

### Random SNP accrual is further confounded by “parallel” evolution

Studies of congenic mice indicate a remarkably high level of parallel evolution (i.e., DNMs observed at the same codon in distinct animal lineages represent approximately 20% of all newly formed SNPs) [2]. Similar evidence of strong parallelism has been noted in human DNA. In one study, for example, 43% of *de novo* SNPs among 1756 trios were found to have occurred more than once when compared to the ExAc mutation dataset [33] (on a ‘random’ basis, <1% of DNMs would have been expected to show recurrence). Among 2,393 patients with CF studied here, 40 exonic SNPs were observed on the F508del background, and at least 5 of these occurred at the same position twice. For intronic DNA encoding 929 mutations on an F508del background, at least 28 overlapped in the same manner. Such findings contradict a notion of “random” DNM production required by dN/dS or pLOF-type analyses, and indicate that other features—such as biased SNP formation—contribute much more prominently than acknowledged previously. Note the stochastic likelihood of observing parallel evolution of the magnitude shown here is infinitesimal. For example, computer simulation of the process indicates that when 969 total SNPs were placed randomly across 184,202 DNA locations (the approximate size of *CFTR*), the number of positions receiving more than one SNP never exceeded 14 when the simulation was conducted 10 million times. Moreover, while two distinct point mutations (e.g., G→A or G→T) at the same codon occur with surprising frequency and can be readily quantified by the present analysis, repeat occurrence of the *same* base replacement at the same position is not easily distinguished from a single mutation simply inherited by multiple individuals (which would only be counted once). In other words, the present analysis is likely to underestimate both frequency and extent of repetitive SNP formation.

As part of the F508del DNA sequence analysis, we also observed pronounced clustering of “parallel” SNPs. For example, in *CFTR* intron 20 (total size 2804 base pairs (bp)), while 59 mutations (29 of these being SNPs) were observed, at least 43 (including 12 SNPs) occurred more than once at the same position (i.e., ≥ 41% of SNPs were found at the same location as a different SNP; Fig 2). Such findings strongly defy random SNP production, and do not result from recent positive selection involving intron 20.

### Origin of the F508del variant

*CFTR* is ancient—over 450 million years old [34]—with thousands of polymorphisms noted in the wildtype protein across numerous ethnicities. Many of the polymorphisms in human *CFTR* may have markedly predated *Homo sapiens* evolution and remain evident because of founder-type effects. The occurrence of F508del on a single *CFTR* haplotype [23, 24, 35, 36] provides compelling evidence for one-time establishment of the variant.

Previous studies using distinct methodology have verified a mutation rate in *Homo sapiens* of approximately 100 SNPs per generation per genome [3, 27, 37–39]. This frequency corresponds to one new SNP per generation for every 30 million bp of human DNA. A total of 1,460 new mutations (~970 SNPs plus small indels, etc.) were noted in the present study when the 184,000-nucleotides of *CFTR* were evaluated across 4,786 alleles (total of 880,624,000 bp). Thirty-eight generations or ~800 years (at 20 years per generation) would be required to achieve the level of diversity in F508del shown here. A similar analysis based on exonic mutations indicates 57 generations (~1,100 years) of F508del SNP accumulation. Note that these timeframe projections may represent a modest underestimate due to consanguinity or other causes of F508del *CFTR* homozygosity, leading to loss of mutant alleles from the gene pool. Moreover, the analysis requires that *CFTR* conforms reasonably well to genome-wide estimates of SNP formation, whereas mutation rates are not yet known with certainty for any individual human gene. In either case, previous estimates place original occurrence of F508del between ~1,100 and 52,000 years ago [23, 36, 40, 41], and our findings support the shorter time line proposed by Serre [41] and Farrell [36]. In the same context, it should be noted that invoking small, epistatic or other recent fitness effects to explain transition, non-synonymous, or parallel mutation preferences on the F508del background over such a short time period would be naive, based on an extensive body of knowledge—and low likelihood that *CFTR* or its role in human physiology have changed substantially over the past few thousand years.

### Evolutionary aspects of *CFTR* adaptation, including significance of the F508del defect

Earlier studies describing cystic fibrosis-related evolutionary biology have focused on CFTR as. a member of the ATP binding cassette (ABC) gene family. Other mammalian ABC proteins utilize ATP hydrolysis as a means to transport solutes across the plasma membrane [11]. *CFTR* has evolved specialized adaptations that include establishment of an ion channel pore, ligand binding interactions, and unique protein kinase A responsive regulatory elements that help govern overall ion channel function. The ways in which these features represent a response to evolutionary pressure have been modeled in part using DNA sequence comparisons with other ABC proteins, such as ABCC4 (in mammals) or YOR1 (in yeast) [42–44]. For example, specific functional divergence of the sixth transmembrane alpha helix at residue R352 has been implicated as contributing to evolutionary establishment of the CFTR permeation pathway. Ancient *CFTR*s such as those encoded by dogfish shark and sea lamprey (>450 million years old) have also facilitated evolutionary inference, including features of both ion channel structure and gating [34]. Some of this earlier work has relied on sequence conservation between species or features of stochastic SNP formation (across much longer time intervals than evaluated here for F508del *CFTR*). The interpretation of such findings might therefore be influenced by features of non-random SNP production as described by the present report.

As another example of previous *CFTR* evolutionary analysis, and in semblance to relationships between sickle cell disease trait and *Plasmodium falciparum*, a heterozygote advantage is believed to account for the high prevalence of F508del *CFTR* carriers in modern-day North America and Europe. It has been argued that a serious infectious disease (e.g., cholera, salmonella, tuberculosis, or plague), or heavy metal poisoning among European populations during the past several thousand years, may have been less lethal among heterozygotes for F508del *CFTR*. Although physiologic and epidemiologic evidence of heterozygote advantage remain inconclusive, the present report shows ways in which the high prevalence of F508del heterozygosity (appx. 1 in 30 White individuals in the US) provides a novel means to evaluate *de novo* SNP accumulation. Our study utilizes the largest compendium of fully sequenced F508del *CFTR* alleles to date–and furnishes a powerful resource for investigating SNP accumulation in human DNA [9, 45–49].

### Limitations of the current approach

Care was taken in the present study to avoid sequencing artifact or incorrect SNP identification by applying a dataset based on state-of-the-art technology and well validated analytic methods (Fig 1 and [26]). SNP prevalence can be subject to ascertainment bias when specific populations (such as patient cohorts that include individuals with cystic fibrosis) are being evaluated, since strong enrichment for “loss of function” alleles is expected. That potential source of bias has been minimized in the current study by focusing on SNPs that occurred subsequent to a CFTR-null (F508del) mutational event. Another potential confounder involves the possibility of second site suppressors that partially rescue F508del CFTR. Known suppressors have been studied by our laboratory and others to characterize CFTR molecular abnormalities [21, 22, 50–54], and if present in cis with F508del could restore CFTR activity. Suppressors of F508del include R1070W, F1068M, F1074M, V510D/E/A, I539T, G550E, R553M/Q, and R555K [21, 22, 50–55]. Importantly, none of these SNPs were observed in the coding sequences obtained from 2,393 patients homozygous for F508del. Additionally, no synonymous SNP in *CFTR* has been reported to rescue the F508del defect. As a result, explaining synonymous SNP enrichment (Table 1) as being caused by numerous synonymous polymorphisms that rescue F508del would appear untenable.

### Adaptive significance of SNP formation bias

It is worth noting that non-random SNP formation as delineated by the current report could provide an important evolutionary advantage by favoring transition, synonymous, and parallel exonic polymorphisms. This mechanism would permit DNA evolutionary adaptiveness and genomic diversity (essential for overcoming environmental pressures and selective “bottlenecks”), while also providing an element of protection for vital DNA protein coding elements and blunting the process of genomic attrition (so-called “mutational meltdown”). Over many hundreds of millions of years (the period of known *CFTR* evolution), an advantage of this type could be substantial [9].

## Conclusions

A large number of reports investigating genomic evolution in *H*. *sapiens* and other species are predicated on an assumption of stochastic *de novo* SNP formation. Hundreds of previous studies have used small changes in dN/dS to draw conclusions that assume “randomness” of DNMs. The F508del haplotype allows a unique test of that premise, and demonstrates strong bias towards *de novo* transition and synonymous polymorphisms, as well as pronounced parallel evolution—all of which complicate an otherwise time-honored strategy for elucidating human adaptation. Taken together with trio and related data indicating considerable variation of DNM frequency and clustering at distinct loci, evolutionary conclusions based on dN/dS, constraint, or related SNP ratios should be carefully scrutinized and may not be valid. The results presented here—in one of the best studied and well characterized human genes—demonstrate SNP formation is not sufficiently random to allow meaningful inference to be drawn using the standard quantitative tools.

## Supporting information

S1 Text Supplemental results. (DOCX)

S1 Fig Positions of exonic *de novo* SNPs in F508del CFTR structural domains. (TIF)

S1 Table SNP distributions across CFTR protein domains. (DOCX)

S1 Appendix Mutation Positions in *CFTR* Python Code. (PDF)

S2 Appendix Codon Frequency in *CFTR* Python Code. (PDF)

S3 Appendix Human *CFTR* sequence dataset. (PDF)

## References

[1] Hill AE, Plyler ZE, Tiwari H, Patki A, Tully JP, McAtee CW, et al. Longevity and plasticity of CFTR provide an argument for noncanonical SNP organization in hominid DNA. PLoS One. 2014;9(10):e109186. Epub 2014/10/29. doi: 10.1371/journal.pone.0109186 ; PubMed Central PMCID: PMC4211684. 25350658 PMC4211684

[2] Plyler ZE, Hill AE, McAtee CW, Cui X, Moseley LA, Sorscher EJ. SNP Formation Bias in the Murine Genome Provides Evidence for Parallel Evolution. Genome Biol Evol. 2015;7(9):2506–19. Epub 2015/08/09. doi: 10.1093/gbe/evv150 ; PubMed Central PMCID: PMC4607513. 26253317 PMC4607513

[3] Michaelson JJ, Shi Y, Gujral M, Zheng H, Malhotra D, Jin X, et al. Whole-genome sequencing in autism identifies hot spots for de novo germline mutation. Cell. 2012;151(7):1431–42. Epub 2012/12/25. doi: 10.1016/j.cell.2012.11.019 ; PubMed Central PMCID: PMC3712641. 23260136 PMC3712641

[4] Habig M, Lorrain C, Feurtey A, Komluski J, Stukenbrock EH. Epigenetic modifications affect the rate of spontaneous mutations in a pathogenic fungus. Nat Commun. 2021;12(1):5869. Epub 20211007. doi: 10.1038/s41467-021-26108-y ; PubMed Central PMCID: PMC8497519. 34620872 PMC8497519

[5] Monroe JG, Srikant T, Carbonell-Bejerano P, Becker C, Lensink M, Exposito-Alonso M, et al. Mutation bias reflects natural selection in Arabidopsis thaliana. Nature. 2022. Epub 20220112. doi: 10.1038/s41586-021-04269-6 . 35022609 PMC8810380

[6] Cano AV, Rozhoňová H, Stoltzfus A, McCandlish DM, Payne JL. Mutation bias shapes the spectrum of adaptive substitutions. Proc Natl Acad Sci U S A. 2022;119(7). doi: 10.1073/pnas.2119720119 ; PubMed Central PMCID: PMC8851560. 35145034 PMC8851560

[7] Cagan A, Baez-Ortega A, Brzozowska N, Abascal F, Coorens THH, Sanders MA, et al. Somatic mutation rates scale with lifespan across mammals. Nature. 2022;604(7906):517–24. Epub 20220413. doi: 10.1038/s41586-022-04618-z ; PubMed Central PMCID: PMC9021023. 35418684 PMC9021023

[8] De Maio N, Walker CR, Turakhia Y, Lanfear R, Corbett-Detig R, Goldman N. Mutation Rates and Selection on Synonymous Mutations in SARS-CoV-2. Genome Biol Evol. 2021;13(5). doi: 10.1093/gbe/evab087 ; PubMed Central PMCID: PMC8135539. 33895815 PMC8135539

[9] Plyler ZE, McAtee CW, Hill AE, Crowley MR, Tindall JM, Tindall SR, et al. Relationships between genomic dissipation and de novo SNP evolution. PLoS One. 2024;19(5):e0303257. Epub 20240516. doi: 10.1371/journal.pone.0303257 . 38753830 PMC11098520

[10] Karczewski KJ, Francioli LC, Tiao G, Cummings BB, Alföldi J, Wang Q, et al. The mutational constraint spectrum quantified from variation in 141,456 humans. Nature. 2020;581(7809):434–43. Epub 2020/05/29. doi: 10.1038/s41586-020-2308-7 ; PubMed Central PMCID: PMC7334197 32461654 PMC7334197

[11] Rowe SM, Miller S, Sorscher EJ. Cystic fibrosis. N Engl J Med. 2005;352(19):1992–2001. Epub 2005/05/13. doi: 10.1056/NEJMra043184 . 15888700

[12] Manfredi C, Tindall JM, Hong JS, Sorscher EJ. Making precision medicine personal for cystic fibrosis. Science. 2019;365(6450):220–1. Epub 2019/07/20. doi: 10.1126/science.aaw0553 ; PubMed Central PMCID: PMC7060931. 31320522 PMC7060931

[13] Yang D, Liang X, Pallas B, Hoenerhoff M, Ren Z, Han R, et al. Production of CFTR-ΔF508 Rabbits. Front Genet. 2020;11:627666. Epub 20210122. doi: 10.3389/fgene.2020.627666 ; PubMed Central PMCID: PMC7862758. 33552140 PMC7862758

[14] Dreano E, Bacchetta M, Simonin J, Galmiche L, Usal C, Slimani L, et al. Characterization of two rat models of cystic fibrosis-KO and F508del CFTR-Generated by Crispr-Cas9. Animal Model Exp Med. 2019;2(4):297–311. Epub 20191125. doi: 10.1002/ame2.12091 ; PubMed Central PMCID: PMC6930998. 31942562 PMC6930998

[15] French PJ, van Doorninck JH, Peters RH, Verbeek E, Ameen NA, Marino CR, et al. A delta F508 mutation in mouse cystic fibrosis transmembrane conductance regulator results in a temperature-sensitive processing defect in vivo. J Clin Invest. 1996;98(6):1304–12. doi: 10.1172/JCI118917 ; PubMed Central PMCID: PMC507556. 8823295 PMC507556

[16] Fisher JT, Liu X, Yan Z, Luo M, Zhang Y, Zhou W, et al. Comparative processing and function of human and ferret cystic fibrosis transmembrane conductance regulator. J Biol Chem. 2012;287(26):21673–85. Epub 20120508. doi: 10.1074/jbc.M111.336537 ; PubMed Central PMCID: PMC3381131. 22570484 PMC3381131

[17] Ostedgaard LS, Meyerholz DK, Chen JH, Pezzulo AA, Karp PH, Rokhlina T, et al. The ΔF508 mutation causes CFTR misprocessing and cystic fibrosis-like disease in pigs. Sci Transl Med. 2011;3(74):74ra24. doi: 10.1126/scitranslmed.3001868 ; PubMed Central PMCID: PMC3119077. 21411740 PMC3119077

[18] Denning GM, Anderson MP, Amara JF, Marshall J, Smith AE, Welsh MJ. Processing of mutant cystic fibrosis transmembrane conductance regulator is temperature-sensitive. Nature. 1992;358(6389):761–4. doi: 10.1038/358761a0 . 1380673

[19] Gregory RJ, Rich DP, Cheng SH, Souza DW, Paul S, Manavalan P, et al. Maturation and function of cystic fibrosis transmembrane conductance regulator variants bearing mutations in putative nucleotide-binding domains 1 and 2. Mol Cell Biol. 1991;11(8):3886–93. doi: 10.1128/mcb.11.8.3886-3893.1991 ; PubMed Central PMCID: PMC361177. 1712898 PMC361177

[20] Dalemans W, Barbry P, Champigny G, Jallat S, Dott K, Dreyer D, et al. Altered chloride ion channel kinetics associated with the delta F508 cystic fibrosis mutation. Nature. 1991;354(6354):526–8. doi: 10.1038/354526a0 . 1722027

[21] Teem JL, Berger HA, Ostedgaard LS, Rich DP, Tsui LC, Welsh MJ. Identification of revertants for the cystic fibrosis delta F508 mutation using STE6-CFTR chimeras in yeast. Cell. 1993;73(2):335–46. doi: 10.1016/0092-8674(93)90233-g . 7682896

[22] Prins S, Corradi V, Sheppard DN, Tieleman DP, Vergani P. Can two wrongs make a right? F508del-CFTR ion channel rescue by second-site mutations in its transmembrane domains. J Biol Chem. 2022;298(3):101615. Epub 20220121. doi: 10.1016/j.jbc.2022.101615 ; PubMed Central PMCID: PMC8861112. 35065958 PMC8861112

[23] Morral N, Bertranpetit J, Estivill X, Nunes V, Casals T, Giménez J, et al. The origin of the major cystic fibrosis mutation (delta F508) in European populations. Nat Genet. 1994;7(2):169–75. doi: 10.1038/ng0694-169 . 7920636

[24] Farrell P. Tracking down the origins of cystic fibrosis in ancient Europe. Smithsonian Magazine. 2018 September 10, 2018.

[25] Sorscher EJ. Cystic fibrosis. In: Jameson JL, et al., editor. Harrison’s Principles of Internal Medicine. 21 ed. New York, NY: McGraw-Hill Education/Medical; 2021.

[26] Raraigh KS, Aksit MA, Hetrick K, Pace RG, Ling H, O’Neal W, et al. Complete CFTR gene sequencing in 5,058 individuals with cystic fibrosis informs variant-specific treatment. J Cyst Fibros. 2022;21(3):463–70. Epub 20211112. doi: 10.1016/j.jcf.2021.10.011 . 34782259

[27] Besenbacher S, Sulem P, Helgason A, Helgason H, Kristjansson H, Jonasdottir A, et al. Multi-nucleotide de novo Mutations in Humans. PLoS Genet. 2016;12(11):e1006315. Epub 2016/11/16. doi: 10.1371/journal.pgen.1006315 ; PubMed Central PMCID: PMC5147774. 27846220 PMC5147774

[28] Bofkin L, Goldman N. Variation in evolutionary processes at different codon positions. Mol Biol Evol. 2007;24(2):513–21. Epub 20061121. doi: 10.1093/molbev/msl178 . 17119011

[29] Freeland SJ, Hurst LD. The genetic code is one in a million. J Mol Evol. 1998;47(3):238–48. Epub 1998/09/11. doi: 10.1007/pl00006381 . 9732450

[30] Iossifov I, Ronemus M, Levy D, Wang Z, Hakker I, Rosenbaum J, et al. De novo gene disruptions in children on the autistic spectrum. Neuron. 2012;74(2):285–99. doi: 10.1016/j.neuron.2012.04.009 ; PubMed Central PMCID: PMC3619976. 22542183 PMC3619976

[31] O’Roak BJ, Vives L, Girirajan S, Karakoc E, Krumm N, Coe BP, et al. Sporadic autism exomes reveal a highly interconnected protein network of de novo mutations. Nature. 2012;485(7397):246–50. Epub 2012/04/13. doi: 10.1038/nature10989 ; PubMed Central PMCID: PMC3350576. 22495309 PMC3350576

[32] Neale BM, Kou Y, Liu L, Ma’ayan A, Samocha KE, Sabo A, et al. Patterns and rates of exonic de novo mutations in autism spectrum disorders. Nature. 2012;485(7397):242–5. Epub 20120404. doi: 10.1038/nature11011 ; PubMed Central PMCID: PMC3613847. 22495311 PMC3613847

[33] Lek M, Karczewski KJ, Minikel EV, Samocha KE, Banks E, Fennell T, et al. Analysis of protein-coding genetic variation in 60,706 humans. Nature. 2016;536(7616):285–91. Epub 2016/08/19. doi: 10.1038/nature19057 ; PubMed Central PMCID: PMC5018207. 27535533 PMC5018207

[34] Cui G, Hong J, Chung-Davidson YW, Infield D, Xu X, Li J, et al. An Ancient CFTR Ortholog Informs Molecular Evolution in ABC Transporters. Dev Cell. 2019;51(4):421–30.e3. Epub 2019/11/05. doi: 10.1016/j.devcel.2019.09.017 ; PubMed Central PMCID: PMC7665244. 31679858 PMC7665244

[35] Vecchio-Pagán B, Blackman SM, Lee M, Atalar M, Pellicore MJ, Pace RG, et al. Deep resequencing of CFTR in 762 F508del homozygotes reveals clusters of non-coding variants associated with cystic fibrosis disease traits. Hum Genome Var. 2016;3:16038. Epub 20161124. doi: 10.1038/hgv.2016.38 ; PubMed Central PMCID: PMC5121184. 27917292 PMC5121184

[36] Farrell P, Férec C, Macek M, Frischer T, Renner S, Riss K, et al. Estimating the age of p.(Phe508del) with family studies of geographically distinct European populations and the early spread of cystic fibrosis. Eur J Hum Genet. 2018;26(12):1832–9. Epub 20180808. doi: 10.1038/s41431-018-0234-z ; PubMed Central PMCID: PMC6244163. 30089827 PMC6244163

[37] Jónsson H, Sulem P, Arnadottir GA, Pálsson G, Eggertsson HP, Kristmundsdottir S, et al. Multiple transmissions of de novo mutations in families. Nat Genet. 2018;50(12):1674–80. Epub 2018/11/07. doi: 10.1038/s41588-018-0259-9 . 30397338

[38] Lynch M. Rate, molecular spectrum, and consequences of human mutation. Proc Natl Acad Sci U S A. 2010;107(3):961–8. Epub 20100104. doi: 10.1073/pnas.0912629107 ; PubMed Central PMCID: PMC2824313. 20080596 PMC2824313

[39] Jobling MA, Tyler-Smith C. The human Y chromosome: an evolutionary marker comes of age. Nat Rev Genet. 2003;4(8):598–612. doi: 10.1038/nrg1124 . 12897772

[40] Fichou Y, Génin E, Le Maréchal C, Audrézet MP, Scotet V, Férec C. Estimating the age of CFTR mutations predominantly found in Brittany (Western France). J Cyst Fibros. 2008;7(2):168–73. Epub 20070906. doi: 10.1016/j.jcf.2007.07.009 . 17825628

[41] Serre JL, Simon-Bouy B, Mornet E, Jaume-Roig B, Balassopoulou A, Schwartz M, et al. Studies of RFLP closely linked to the cystic fibrosis locus throughout Europe lead to new considerations in populations genetics. Hum Genet. 1990;84(5):449–54. doi: 10.1007/BF00195818 . 1969843

[42] Oliver KE, Rauscher R, Mijnders M, Wang W, Wolpert MJ, Maya J, et al. Slowing ribosome velocity restores folding and function of mutant CFTR. J Clin Invest. 2019;129(12):5236–53. Epub 2019/10/29. doi: 10.1172/JCI124282 ; PubMed Central PMCID: PMC6877332. 31657788 PMC6877332

[43] Jordan IK, Kota KC, Cui G, Thompson CH, McCarty NA. Evolutionary and functional divergence between the cystic fibrosis transmembrane conductance regulator and related ATP-binding cassette transporters. Proc Natl Acad Sci U S A. 2008;105(48):18865–70. Epub 20081119. doi: 10.1073/pnas.0806306105 ; PubMed Central PMCID: PMC2585040. 19020075 PMC2585040

[44] Infield DT, Strickland KM, Gaggar A, McCarty NA. The molecular evolution of function in the CFTR chloride channel. J Gen Physiol. 2021;153(12). Epub 20211014. doi: 10.1085/jgp.202012625 ; PubMed Central PMCID: PMC8640958. 34647973 PMC8640958

[45] Poolman EM, Galvani AP. Evaluating candidate agents of selective pressure for cystic fibrosis. J R Soc Interface. 2007;4(12):91–8. Epub 2006/10/04. doi: 10.1098/rsif.2006.0154 ; PubMed Central PMCID: PMC2358959. 17015291 PMC2358959

[46] Gabriel SE, Brigman KN, Koller BH, Boucher RC, Stutts MJ. Cystic fibrosis heterozygote resistance to cholera toxin in the cystic fibrosis mouse model. Science. 1994;266(5182):107–9. Epub 1994/10/07. doi: 10.1126/science.7524148 . 7524148

[47] Pier GB, Grout M, Zaidi T, Meluleni G, Mueschenborn SS, Banting G, et al. Salmonella typhi uses CFTR to enter intestinal epithelial cells. Nature. 1998;393(6680):79–82. Epub 1998/05/20. doi: 10.1038/30006 . 9590693

[48] Bosch L, Bosch B, De Boeck K, Nawrot T, Meyts I, Vanneste D, et al. Cystic fibrosis carriership and tuberculosis: hints toward an evolutionary selective advantage based on data from the Brazilian territory. BMC Infect Dis. 2017;17(1):340. Epub 2017/05/14. doi: 10.1186/s12879-017-2448-z ; PubMed Central PMCID: PMC5429554. 28499359 PMC5429554

[49] Cuthbert AW, Halstead J, Ratcliff R, Colledge WH, Evans MJ. The genetic advantage hypothesis in cystic fibrosis heterozygotes: a murine study. J Physiol. 1995;482 (Pt 2)(Pt 2):449–54. Epub 1995/01/15. doi: 10.1113/jphysiol.1995.sp020531 ; PubMed Central PMCID: PMC1157742. 7714835 PMC1157742

[50] Loo TW, Bartlett MC, Clarke DM. The V510D suppressor mutation stabilizes DeltaF508-CFTR at the cell surface. Biochemistry. 2010;49(30):6352–7. doi: 10.1021/bi100807h ; PubMed Central PMCID: PMC2911077. 20590134 PMC2911077

[51] Ren HY, Grove DE, De La Rosa O, Houck SA, Sopha P, Van Goor F, et al. VX-809 corrects folding defects in cystic fibrosis transmembrane conductance regulator protein through action on membrane-spanning domain 1. Mol Biol Cell. 2013;24(19):3016–24. Epub 20130807. doi: 10.1091/mbc.E13-05-0240 ; PubMed Central PMCID: PMC3784376. 23924900 PMC3784376

[52] Wang Y, Loo TW, Bartlett MC, Clarke DM. Correctors promote maturation of cystic fibrosis transmembrane conductance regulator (CFTR)-processing mutants by binding to the protein. J Biol Chem. 2007;282(46):33247–51. Epub 20071002. doi: 10.1074/jbc.C700175200 . 17911111

[53] DeCarvalho AC, Gansheroff LJ, Teem JL. Mutations in the nucleotide binding domain 1 signature motif region rescue processing and functional defects of cystic fibrosis transmembrane conductance regulator delta f508. J Biol Chem. 2002;277(39):35896–905. Epub 20020710. doi: 10.1074/jbc.M205644200 . 12110684

[54] He L, Aleksandrov LA, Cui L, Jensen TJ, Nesbitt KL, Riordan JR. Restoration of domain folding and interdomain assembly by second-site suppressors of the DeltaF508 mutation in CFTR. Faseb j. 2010;24(8):3103–12. Epub 20100316. doi: 10.1096/fj.09-141788 ; PubMed Central PMCID: PMC2909275. 20233947 PMC2909275

[55] Teem JL, Carson MR, Welsh MJ. Mutation of R555 in CFTR-delta F508 enhances function and partially corrects defective processing. Recept Channels. 1996;4(1):63–72. . 8723647

