# Supplementary material for: Mutation accumulation in H. sapiens F508del CFTR countermands dN/dS type genomic analysis
Source: PLoS One. 2024 Jul 18;19(7):e0305832. doi: 10.1371/journal.pone.0305832 (PMC11257350; doi:10.1371/journal.pone.0305832)
Supplement: S1 Text — (DOCX) [file pone.0305832.s001.docx]

# **Supporting Information**

**S1 Text: Supplemental results**

While deleterious *CFTR* variants among patients with cystic fibrosis may be modestly increased within certain structural domains of the protein (e.g., TMD1, NBD1), this is due in part to ascertainment bias (favoring protein-disruptive mutations when studied specifically in patients with the disease). In contrast, the gnomAD database uses unbiased analysis and has identified over 3,000 exonic SNPs that are well represented throughout the five primary *CFTR* structural domains (S1 Table). Similarly, *de novo* SNPs identified from F508del alleles in the present study reflect minimal ascertainment bias, and do not exhibit strong preferences for any *CFTR* domain (S1 Fig).
