# Supplementary figures and images for: Mutation accumulation in H. sapiens F508del CFTR countermands dN/dS type genomic analysis
Source: PLoS One. 2024 Jul 18;19(7):e0305832. doi: 10.1371/journal.pone.0305832 (PMC11257350; doi:10.1371/journal.pone.0305832)

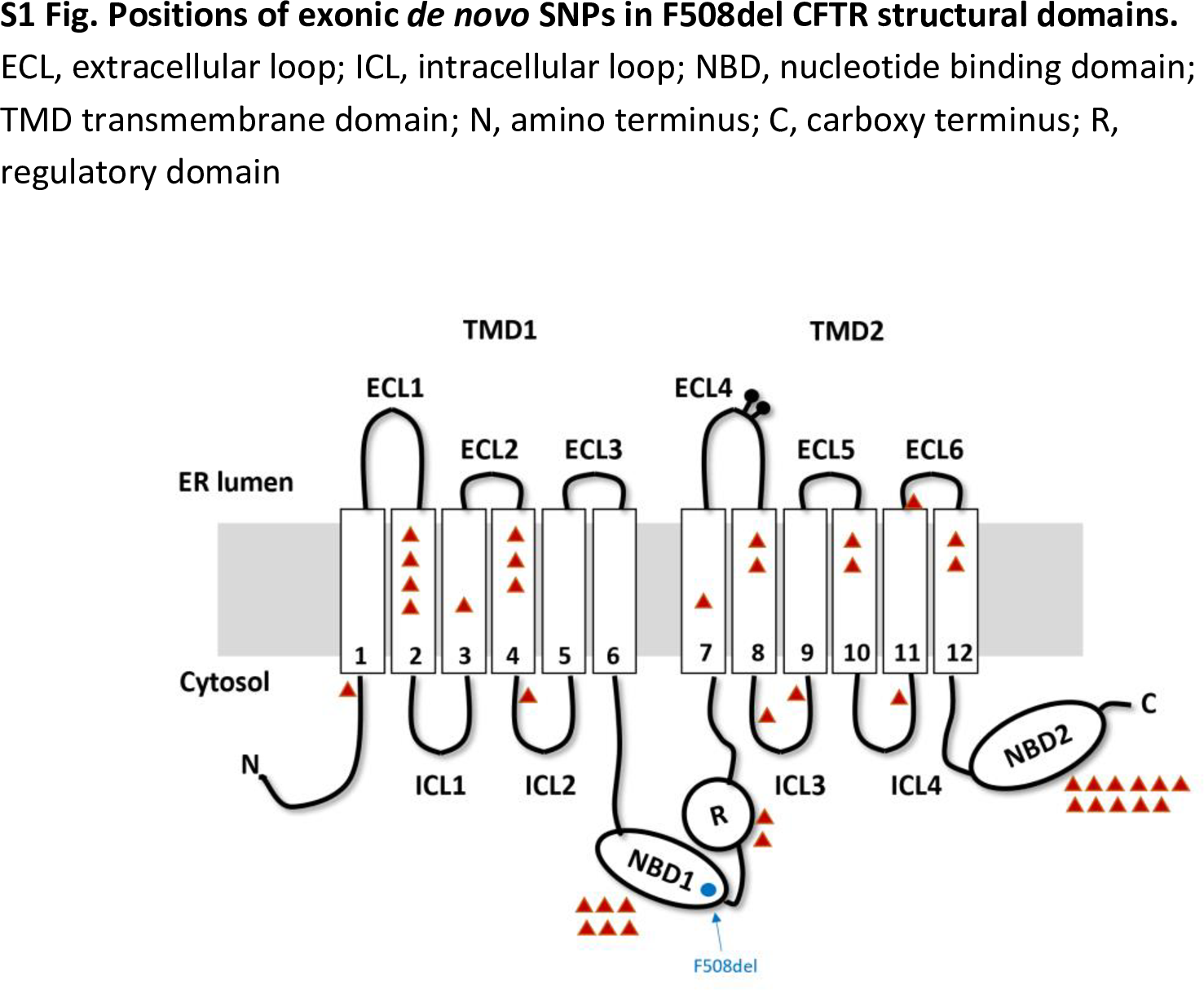

Supplement: S1 Fig — (TIF) [file pone.0305832.s002.tif]
