# Supplementary material for: Mutation accumulation in H. sapiens F508del CFTR countermands dN/dS type genomic analysis
Source: PLoS One. 2024 Jul 18;19(7):e0305832. doi: 10.1371/journal.pone.0305832 (PMC11257350; doi:10.1371/journal.pone.0305832)
Supplement: S1 Table — (DOCX) [file pone.0305832.s003.docx]

**S1 Table**. **SNP distributions across CFTR protein domains.** *De novo* mutations identified using unbiased analysis of F508del alleles, and control studies from >100,000 individuals monitored by gnomAD[10] are shown. Strong domain enrichment was not observed with either dataset (i.e., SNPs are well distributed throughout full length *CFTR*). It is difficult to directly compare the two SNP collections, since gnomAD represents multiple ethnicities worldwide, numerous distinct “founder effects,” identity by descent, etc., whereas F508del is believed to have occurred on a single DNA background. NBD, nucleotide binding domain; TMD transmembrane domain; N, amino terminus; C, carboxy terminus; R, regulatory domain; aa, amino acid

|  | # aa in domain | # SNPs in *CFTR* coding sequences | |
| --- | --- | --- | --- |
|  |  | F508del background | gnomAD |
| N-terminal domain (1-79) | 79 | 1 | 189 |
| TMD1 (80-389) | 310 | 9 | 744 |
| NBD1 (390-673) | 284 | 6 | 598 |
| R-domain (674-829) | 156 | 2 | 317 |
| TMD 2 (830-1200) | 371 | 11 | 751 |
| NBD2 (1201-1480) | 280 | 11 | 567 |
| SUM | 1480 | 40 | 3166 |

|  | % total aa in  domain | SNP % in *CFTR* coding sequences | |
| --- | --- | --- | --- |
|  |  | F508del background | gnomAD |
| N-terminal domain (1-79) | 5.34% | 2.50% | 5.97% |
| TMD1 (80-389) | 20.95% | 22.50% | 23.50% |
| NBD1 (390-673) | 19.19% | 15.00% | 18.89% |
| R-domain (674-829) | 10.54% | 5.00% | 10.01% |
| TMD 2 (830-1200) | 25.07% | 27.50% | 23.72% |
| NBD2 (1201-1480) | 18.92% | 27.50% | 17.91% |
| SUM | 100% | 100% | 100% |
