# Supplementary material for: Mutation accumulation in H. sapiens F508del CFTR countermands dN/dS type genomic analysis
Source: PLoS One. 2024 Jul 18;19(7):e0305832. doi: 10.1371/journal.pone.0305832 (PMC11257350; doi:10.1371/journal.pone.0305832)
Supplement: S1 Appendix — (PDF) [file pone.0305832.s004.pdf]

```
import random

#region Constants
#The number of places a mutation could occur, corresponding to the total size of the CFTR gene
POSSIBLE_LOCATIONS = 184202
#The number of mutations that we are putting in per simulation
MUTATIONS = 969
#The number of times we are simulating the process
SIMULATIONS = 10000000
#endregion

#A simple function to calculate the average later
def Average(lst):
    return sum(lst) / len(lst)

#Creating an empty list to store how many duplicate mutations we get at the same position for each simulation
duplicates_count = []

#Creating a variable to hold the random position at which a mutation will occur
index = 0
#Loop that will run for the desired number of simulations, according to the value put into the constant
for s in range(SIMULATIONS):
    #Creating a list of 0s with the length of the CFTR gene, done each loop to reset from the previous simulation
    positions = [0] * POSSIBLE_LOCATIONS
    #Loop that will run the desired number of mutations per simulation, according to the value put into the constant
    for b in range(MUTATIONS):
        #Determining a random location to put the mutation somewhere along the CFTR gene
        index = random.randrange(0, POSSIBLE_LOCATIONS)
        #Increment the number of times that position has received a mutation
        #Because the only thing that matters is if a position received more than 1 mutation, we are only incrementing if the current value is 0 or 1
        if positions[index] < 2:
            positions[index] += 1
    #Count the number of positions where at least 2 mutations occurred, then clear the list to run another simulation
    duplicates_count.append(positions.count(2))
    positions.clear()

#Output the results to the console
print("Simulated " + str(SIMULATIONS) + " times.")
print("Highest value: " + str(max(duplicates_count)))
print("Average number: " + str(Average(duplicates_count)))
```
