# Supplementary material for: Mutation accumulation in H. sapiens F508del CFTR countermands dN/dS type genomic analysis
Source: PLoS One. 2024 Jul 18;19(7):e0305832. doi: 10.1371/journal.pone.0305832 (PMC11257350; doi:10.1371/journal.pone.0305832)
Supplement: S2 Appendix — (PDF) [file pone.0305832.s005.pdf]

```
from textwrap import wrap
import collections
import csv

#region Constants
#Establising each of the possible combinations of 3 amino acids
CODONS = ["TTT", "TTC", "TTA", "TTG", "TCT", "TCC", "TCA", "TCG", "TAT", "TAC", "TAA",
"TAG", "TGT", "TGC", "TGA", "TGG" , "CTT", "CTC", "CTA", "CTG", "CCT", "CCC", "CCA",
"CCG", "CAT", "CAC", "CAA", "CAG", "CGT", "CGC", "CGA", "CGG" , "ATT", "ATC", "ATA",
"ATG", "ACT", "ACC", "ACA", "ACG", "AAT", "AAC", "AAA", "AAG", "AGT", "AGC", "AGA",
"AGG" , "GTT", "GTC", "GTA", "GTG", "GCT", "GCC", "GCA", "GCG", "GAT", "GAC", "GAA",
"GAG", "GGT", "GGC", "GGA", "GGG"]

#The number of times each codon appears across the human genome per 1000 codons,
according to https://www.genscript.com/tools/codon-frequency-table
#This will be used as a reference to compare the frequencies of each codon within the CFTR
gene
FREQUENCY = {
	"TTT": 16.9,
	"TTC": 20.4,
	"TTA": 7.2,
	"TTG": 12.6,
	"TCT": 14.6,
	"TCC": 17.4,
	"TCA": 11.7,
	"TCG": 4.5,
	"TAT": 12.0,
	"TAC": 15.6,
	"TAA": 0.7,
	"TAG": 0.5,
	"TGT": 9.9,
	"TGC": 12.2,
	"TGA": 1.3,
	"TGG": 12.8,
	"CTT": 12.8,
	"CTC": 19.4,
	"CTA": 6.9,
	"CTG": 40.3,
	"CCT": 17.3,
	"CCC": 20.0,
	"CCA": 16.7,
	"CCG": 7.0,
	"CAT": 10.4,
	"CAC": 14.9,
	"CAA": 11.8,
	"CAG": 34.6,
	"CGT": 4.7,
```

```
        "CGC": 10.9,
        "CGA": 6.3,
        "CGG": 11.9,
        "ATT": 15.7,
        "ATC": 21.4,
        "ATA": 7.1,
        "ATG": 22.3,
        "ACT": 12.8,
        "ACC": 19.2,
        "ACA": 14.8,
        "ACG": 6.2,
        "AAT": 16.7,
        "AAC": 19.5,
        "AAA": 24.0,
        "AAG": 32.9,
        "AGT": 11.9,
        "AGC": 19.4,
        "AGA": 11.5,
        "AGG": 11.4,
        "GTT": 10.9,
        "GTC": 14.6,
        "GTA": 7.0,
        "GTG": 28.9,
        "GCT": 18.6,
        "GCC": 28.5,
        "GCA": 16.0,
        "GCG": 7.6,
        "GAT": 22.3,
        "GAC": 26.0,
        "GAA": 29.0,
        "GAG": 40.8,
        "GGT": 10.8,
        "GGC": 22.8,
        "GGA": 16.3,
        "GGG": 16.4}

#Amino acid sequence for CFTR
CFTR_SEQUENCE =
"ATGCAGAGGTCGCCTCTGGAAAAGGCCAGCGTTGTCTCCAAACTTTTTTTCAGCTGGACC
AGACCAATTTTGAGGAAAGGATACAGACAGCGCCTGGAATTGTCAGACATATACCAAATCC
CTTCTGTTGATTCTGCTGACAATCTATCTGAAAAATTGGAAAGAGAATGGGATAGAGAGCT
GGCTTCAAAGAAAAATCCTAAACTCATTAATGCCCTTCGGCGATGTTTTTTCTGGAGATTTA
TGTTCTATGGAATCTTTTTATATTTAGGGGAAGTCACCAAAGCAGTACAGCCTCTCTTACTG
GGAAGAATCATAGCTTCCTATGACCCGGATAACAAGGAGGAACGCTCTATCGCGATTTATC
TAGGCATAGGCTTATGCCTTCTCTTTATTGTGAGGACACTGCTCCTACACCCAGCCATTTTT
GGCCTTCATCACATTGGAATGCAGATGAGAATAGCTATGTTTAGTTTGATTTATAAGAAGAC
TTTAAAGCTGTCAAGCCGTGTTCTAGATAAAATAAGTATTGGACAACTTGTTAGTCTCCTTT
CCAACAACCTGAACAAATTTGATGAAGGACTTGCATTGGCACATTTCGTGTGGATCGCTCC
```

TTTGCAAGTGGCACTCCTCATGGGGCTAATCTGGGAGTTGTTACAGGCGTCTGCCTTCTGT
GGACTTGGTTTCCTGATAGTCCTTGCCCTTTTTCAGGCTGGGCTAGGGAGAATGATGATGA
AGTACAGAGATCAGAGAGCTGGGAAGATCAGTGAAAGACTTGTGATTACCTCAGAAATGAT
TGAAAATATCCAATCTGTTAAGGCATACTGCTGGGAAGAAGCAATGGAAAAAATGATTGAAA
ACTTAAGACAAACAGAACTGAAACTGACTCGGAAGGCAGCCTATGTGAGATACTTCAATAG
CTCAGCCTTCTTCTTCTCAGGGTTCTTTGTGGTGTTTTTATCTGTGCTTCCCTATGCACTAAT
CAAAGGAATCATCCTCCGGAAAATATTCACCACCATCTCATTCTGCATTGTTCTGCGCATGG
CGGTCACTCGGCAATTTCCCTGGGCTGTACAAACATGGTATGACTCTCTTGGAGCAATAAA
CAAAATACAGGATTTCTTACAAAAGCAAGAATATAAGACATTGGAATATAACTTAACGACTA
CAGAAGTAGTGATGGAGAATGTAACAGCCTTCTGGGAGGAGGGATTTGGGGAATTATTTGA
GAAAGCAAAACAAAACAATAACAATAGAAAAACTTCTAATGGTGATGACAGCCTCTTCTTCA
GTAATTTCTCACTTCTTGGTACTCCTGTCCTGAAAGATATTAATTTCAAGATAGAAAGAGGA
CAGTTGTTGGCGGTTGCTGGATCCACTGGAGCAGGCAAGACTTCACTTCTAATGATGATTA
TGGGAGAACTGGAGCCTTCAGAGGGTAAAATTAAGCACAGTGGAAGAATTTCATTCTGTTC
TCAGTTTTCCTGGATTATGCCTGGCACCATTAAAGAAAATATCATCTTTGGTGTTTCCTATG
ATGAATATAGATACAGAAGCGTCATCAAAGCATGCCAACTAGAAGAGGACATCTCCAAGTT
TGCAGAGAAAGACAATATAGTTCTTGGAGAAGGTGGAATCACACTGAGTGGAGGTCAACG
AGCAAGAATTTCTTTAGCAAGAGCAGTATACAAAGATGCTGATTTGTATTTATTAGACTCTC
CTTTTGGATACCTAGATGTTTTAACAGAAAAAGAAATATTTGAAAGCTGTGTCTGTAAACTG
ATGGCTAACAAAACTAGGATTTTGGTCACTTCTAAAATGGAACATTTAAAGAAAGCTGACAA
AATATTAATTTTGCATGAAGGTAGCAGCTATTTTTATGGGACATTTTCAGAACTCCAAAATCT
ACAGCCAGACTTTAGCTCAAAACTCATGGGATGTGATTCTTTCGACCAATTTAGTGCAGAAA
GAAGAAATTCAATCCTAACTGAGACCTTACACCGTTTCTCATTAGAAGGAGATGCTCCTGTC
TCCTGGACAGAAACAAAAAAACAATCTTTTAAACAGACTGGAGAGTTTGGGGAAAAAAGGA
AGAATTCTATTCTCAATCCAATCAACTCTATACGAAAATTTTCCATTGTGCAAAAGACTCCCT
TACAAATGAATGGCATCGAAGAGGATTCTGATGAGCCTTTAGAGAGAAGGCTGTCCTTAGT
ACCAGATTCTGAGCAGGGAGAGGCGATACTGCCTCGCATCAGCGTGATCAGCACTGGCCC
CACGCTTCAGGCACGAAGGAGGCAGTCTGTCCTGAACCTGATGACACACTCAGTTAACCA
AGGTCAGAACATTCACCGAAAGACAACAGCATCCACACGAAAAGTGTCACTGGCCCCTCA
GGCAAACTTGACTGAACTGGATATATATTCAAGAAGGTTATCTCAAGAAACTGGCTTGGAAA
TAAGTGAAGAAATTAACGAAGAAGACTTAAAGGAGTGCTTTTTTGATGATATGGAGAGCATA
CCAGCAGTGACTACATGGAACACATACCTTCGATATATTACTGTCCACAAGAGCTTAATTTT
TGTGCTAATTTGGTGCTTAGTAATTTTTCTGGCAGAGGTGGCTGCTTCTTTGGTTGTGCTGT
GGCTCCTTGGAAACACTCCTCTTCAAGACAAAGGGAATAGTACTCATAGTAGAAATAACAG
CTATGCAGTGATTATCACCAGCACCAGTTCGTATTATGTGTTTTACATTTACGTGGGAGTAG
CCGACACTTTGCTTGCTATGGGATTCTTCAGAGGTCTACCACTGGTGCATACTCTAATCAC
AGTGTCGAAAATTTTACACCACAAAATGTTACATTCTGTTCTTCAAGCACCTATGTCAACCC
TCAACACGTTGAAAGCAGGTGGGATTCTTAATAGATTCTCCAAAGATATAGCAATTTTGGAT
GACCTTCTGCCTCTTACCATATTTGACTTCATCCAGTTGTTATTAATTGTGATTGGAGCTATA
GCAGTTGTCGCAGTTTTACAACCCTACATCTTTGTTGCAACAGTGCCAGTGATAGTGGCTTT
TATTATGTTGAGAGCATATTTCCTCCAAACCTCACAGCAACTCAAACAACTGGAATCTGAAG
GCAGGAGTCCAATTTTCACTCATCTTGTTACAAGCTTAAAAGGACTATGGACACTTCGTGCC
TTCGGACGGCAGCCTTACTTTGAAACTCTGTTCCACAAAGCTCTGAATTTACATACTGCCAA
CTGGTTCTTGTACCTGTCAACACTGCGCTGGTTCCAAATGAGAATAGAAATGATTTTTGTCA
TCTTCTTCATTGCTGTTACCTTCATTTCCATTTTAACAACAGGAGAAGGAGAAGGAAGAGTT
GGTATTATCCTGACTTTAGCCATGAATATCATGAGTACATTGCAGTGGGCTGTAAACTCCAG
CATAGATGTGGATAGCTTGATGCGATCTGTGAGCCGAGTCTTTAAGTTCATTGACATGCCA
ACAGAAGGTAAACCTACCAAGTCAACCAAACCATACAAGAATGGCCAACTCTCGAAAGTTA
TGATTATTGAGAATTCACACGTGAAGAAAGATGACATCTGGCCCTCAGGGGGCCAAATGAC
TGTCAAAGATCTCACAGCAAAATACACAGAAGGTGGAAATGCCATATTAGAGAACATTTCCT
TCTCAATAAGTCCTGGCCAGAGGGTGGGCCTCTTGGGAAGAACTGGATCAGGGAAGAGTA

```
CTTTGTTATCAGCTTTTTTGAGACTACTGAACACTGAAGGAGAAATCCAGATCGATGGTGTG
TCTTGGGATTCAATAACTTTGCAACAGTGGAGGAAAGCCTTTGGAGTGATACCACAGAAAG
TATTTATTTTTTCTGGAACATTTAGAAAAAACTTGGATCCCTATGAACAGTGGAGTGATCAA
GAAATATGGAAAGTTGCAGATGAGGTTGGGCTCAGATCTGTGATAGAACAGTTTCCTGGGA
AGCTTGACTTTGTCCTTGTGGATGGGGGCTGTGTCCTAAGCCATGGCCACAAGCAGTTGAT
GTGCTTGGCTAGATCTGTTCTCAGTAAGGCGAAGATCTTGCTGCTTGATGAACCCAGTGCT
CATTTGGATCCAGTAACATACCAAATAATTAGAAGAACTCTAAAACAAGCATTTGCTGATTG
CACAGTAATTCTCTGTGAACACAGGATAGAAGCAATGCTGGAATGCCAACAATTTTTGGTC
ATAGAAGAGAACAAAGTGCGGCAGTACGATTCCATCCAGAAACTGCTGAACGAGAGGAGC
CTCTTCCGGCAAGCCATCAGCCCCTCCGACAGGGTGAAGCTCTTTCCCCACCGGAACTCA
AGCAAGTGCAAGTCTAAGCCCCAGATTGCTGCTCTGAAAGAGGAGACAGAAGAAGAGGTG
CAAGATACAAGGCTTTAG"
#endregion

#Please input a valid .csv file path here if you wish to run the code for yourself
#For example, "c:/Users/YourNameHere/Documents/codonFrequencyCFTR.csv"
outputFileName = ""

#The frequency of codons per 1000 may have rounding errors due to precision, so we start by
finding the total (which comes out to 999.5)
total_frequency = 0
for c in FREQUENCY:
	total_frequency += FREQUENCY[c]

#We take the full CFTR sequence and break it into a list of 3 character strings corresponding to
each codon
cftrCodons = wrap(CFTR_SEQUENCE, 3)
#In order to calculate frequency within the CFTR gene, we need the total number of codons in
the sequence
cftrTotal = len(cftrCodons)
#Using collections.Counter to make a dictionary where each codon is stored with the number of
times it occurs in the list of codons
cftrFrequency = dict(collections.Counter(cftrCodons))

#Setting up headers for an output csv file;
#"occurances (cftr)" is the number of times that the corresponding codon occured in the CFTR
sequence
#"relative frequency (cftr)" columns will be used to store the portion of all codons made up by
that specific codon (i.e. occurances of that codon divided by the total number of codons)
#"occurances (general)" is likely a useless column since "occurances (cftr)" is not in per 1000
codons, but it was left in just in case
#"relative frequency (general)" is like the cftr counterpart, but using the frequency information for
the whole human genome
header = ["codon", "occurances (cftr)", "relative frequency (cftr)", "occurances (general)",
"relative frequency (general)"]
#Please remember to input a valid file path in the variable above
f = open(outputFileName, "w")
writer = csv.writer(f)
writer.writerow(header)
```

```
#Time to start putting everything into a csv file with a row for every possible codon, which can
be easily copied to another spreadsheet for additional analysis for c in CODONS:
	if c in cftrFrequency:
		#We write in the data needed for each column, as explained above
		writer.writerow([c, cftrFrequency[c], cftrFrequency[c]/cftrTotal, FREQUENCY[c],
	FREQUENCY[c]/total_frequency])
		else:
		#Not all possible codons appear in CFTR, so those occur 0 times
		writer.writerow([c, 0, 0, FREQUENCY[c], FREQUENCY[c]/total_frequency])

f.close()
```
