# Supplementary material for: Mutation accumulation in H. sapiens F508del CFTR countermands dN/dS type genomic analysis
Source: PLoS One. 2024 Jul 18;19(7):e0305832. doi: 10.1371/journal.pone.0305832 (PMC11257350; doi:10.1371/journal.pone.0305832)
Supplement: S3 Appendix — (PDF) [file pone.0305832.s006.pdf]

Human CFTR

1 ATGCAGAGGTCGCCT CTGGAAAAGGCCAGC GTTGTCTCCAAACTT TTTTTCAGCTGGACC
1 M Q R S P L E K A S V V S K L F F S W T

61 AGACCAATTTTGAGG AAAGGATACAGACAG CGCCTGGAATTGTCA GACATATACCAAATC
21 R P I L R K G Y R Q R L E L S D I Y Q I

121 CCTTCTGTTGATTCT GCTGACAATCTATCT GAAAAATTGGAAAGA GAATGGGATAGAGAG
41 P S V D S A D N L S E K L E R E W D R E

181 CTGGCTTCAAAGAAA AATCCTAAACTCATT AATGCCCTTCGGCGA TGTTTTTTCTGGAGA
61 L A S K K N P K L I N A L R R C F F W R

241 TTTATGTTCTATGGA ATCTTTTTATATTTA GGGGAAGTCACCAAA GCAGTACAGCCTCTC
81 F M F Y G I F L Y L G E V T K A V Q P L

301 TTACTGGGAAGAATC ATAGCTTCCTATGAC CCGGATAACAAGGAG GAACGCTCTATCGCG
101 L L G R I I A S Y D P D N K E E R S I A

361 ATTTATCTAGGCATA GGCTTATGCCTTCTC TTTATTGTGAGGACA CTGCTCCTACACCCA
121 I Y L G I G L C L L F I V R T L L L H P

421 GCCATTTTTGGCCTT CATCACATTGGAATG CAGATGAGAATAGCT ATGTTTAGTTTGATT
141 A I F G L H H I G M Q M R I A M F S L I

481 TATAAGAAGACTTTA AAGCTGTCAAGCCGT GTTCTAGATAAAATA AGTATTGGACAACTT
161 Y K K T L K L S S R V L D K I S I G Q L

541 GTTAGTCTCCTTTCC AACAACCTGAACAAA TTTGATGAAGGACTT GCATTGGCACATTTC
181 V S L L S N N L N K F D E G L A L A H F

601 GTGTGGATCGCTCCT TTGCAAGTGGCACTC CTCATGGGGCTAATC TGGGAGTTGTTACAG
201 V W I A P L Q V A L L M G L I W E L L Q

661 GCGTCTGCCTTCTGT GGACTTGGTTTCCTG ATAGTCCTTGCCCTT TTTCAGGCTGGGCTA
221 A S A F C G L G F L I V L A L F Q A G L

721 GGGAGAATGATGATG AAGTACAGAGATCAG AGAGCTGGGAAGATC AGTGAAAGACTTGTG
241 G R M M M K Y R D Q R A G K I S E R L V

781 ATTACCTCAGAAATG ATTGAAAATATCCAA TCTGTTAAGGCATAC TGCTGGGAAGAAGCA
261 I T S E M I E N I Q S V K A Y C W E E A

841 ATGGAAAAAATGATT GAAAACTTAAGACAA ACAGAACTGAAACTG ACTCGGAAGGCAGCC
281 M E K M I E N L R Q T E L K L T R K A A

901 TATGTGAGATACTTC AATAGCTCAGCCTTC TTCTTCTCAGGGTTC TTTGTGGTGTTTTTA
301 Y V R Y F N S S A F F F S G F F V V F L

961	TCTGTGCTTCCCTAT	GCACTAATCAAAGGA	ATCATCCTCCGGAAA	ATATTCACCACCATC
321	S V L P Y	A L I K G	I I L R K	I F T T I
1021	TCATTCTGCATTGTT	CTGCGCATGGCGGTC	ACTCGGCAATTTCCC	TGGGCTGTACAAACA
341	S F C I V	L R M A V	T R Q F P	W A V Q T
1081	TGGTATGACTCTCTT	GGAGCAATAAACAAA	ATACAGGATTTCTTA	CAAAAGCAAGAATAT
361	W Y D S L	G A I N K	I Q D F L	Q K Q E Y
1141	AAGACATTGGAATAT	AACTTAACGACTACA	GAAGTAGTGATGGAG	AATGTAACAGCCTTC
381	K T L E Y	N L T T T	E V V M E	N V T A F
1201	TGGGAGGAGGGATTT	GGGGAATTATTTGAG	AAAGCAAAACAAAAC	AATAACAATAGAAAA
401	W E E G F	G E L F E	K A K Q N	N N N R K
1261	ACTTCTAATGGTGAT	GACAGCCTCTTCTTC	AGTAATTTCTCACTT	CTTGGTACTCCTGTC
421	T S N G D	D S L F F	S N F S L	L G T P V
1321	CTGAAAGATATTAAT	TTCAAGATAGAAAGA	GGACAGTTGTTGGCG	GTTGCTGGATCCACT
441	L K D I N	F K I E R	G Q L L A	V A G S T
1381	GGAGCAGGCAAGACT	TCACTTCTAATGATG	ATTATGGGAGAACTG	GAGCCTTCAGAGGGT
461	G A G K T	S L L M M	I M G E L	E P S E G
1441	AAAATTAAGCACAGT	GGAAGAATTTCATTC	TGTTCTCAGTTTTCC	TGGATTATGCCTGGC
481	K I K H S	G R I S F	C S Q F S	W I M P G
1501	ACCATTAAAGAAAAT	ATCATCTTTGGTGTT	TCCTATGATGAATAT	AGATACAGAAGCGTC
501	T I K E N	I I F G V	S Y D E Y	R Y R S V
1561	ATCAAAGCATGCCAA	CTAGAAGAGGACATC	TCCAAGTTTGCAGAG	AAAGACAATATAGTT
521	I K A C Q	L E E D I	S K F A E	K D N I V
1621	CTTGGAGAAGGTGGA	ATCACACTGAGTGGA	GGTCAACGAGCAAGA	ATTTCTTTAGCAAGA
541	L G E G G	I T L S G	G Q R A R	I S L A R
1681	GCAGTATACAAAGAT	GCTGATTTGTATTTA	TTAGACTCTCCTTTT	GGATACCTAGATGTT
561	A V Y K D	A D L Y L	L D S P F	G Y L D V
1741	TTAACAGAAAAAGAA	ATATTTGAAAGCTGT	GTCTGTAAACTGATG	GCTAACAAAACTAGG
581	L T E K E	I F E S C	V C K L M	A N K T R
1801	ATTTTGGTCACTTCT	AAAATGGAACATTTA	AAGAAAGCTGACAAA	ATATTAATTTTGCAT
601	I L V T S	K M E H L	K K A D K	I L I L H
1861	GAAGGTAGCAGCTAT	TTTTATGGGACATTT	TCAGAACTCCAAAAT	CTACAGCCAGACTTT
621	E G S S Y	F Y G T F	S E L Q N	L Q P D F

1921	AGCTCAAAACTCATG	GGATGTGATTCTTTC	GACCAATTTAGTGCA	GAAAGAAGAAATTCA
641	S S K L M	G C D S F	D Q F S A	E R R N S
1981	ATCCTAACTGAGACC	TTACACCGTTTCTCA	TTAGAAGGAGATGCT	CCTGTCTCCTGGACA
661	I L T E T	L H R F S	L E G D A	P V S W T
2041	GAAACAAAAAAACAA	TCTTTTAAACAGACT	GGAGAGTTTGGGGAA	AAAAGGAAGAATTCT
681	E T K K Q	S F K Q T	G E F G E	K R K N S
2101	ATTCTCAATCCAATC	AACTCTATACGAAAA	TTTTCCATTGTGCAA	AAGACTCCCTTACAA
701	I L N P I	N S I R K	F S I V Q	K T P L Q
2161	ATGAATGGCATCGAA	GAGGATTCTGATGAG	CCTTTAGAGAGAAGG	CTGTCCTTAGTACCA
721	M N G I E	E D S D E	P L E R R	L S L V P
2221	GATTCTGAGCAGGGA	GAGGCGATACTGCCT	CGCATCAGCGTGATC	AGCACTGGCCCCACG
741	D S E Q G	E A I L P	R I S V I	S T G P T
2281	CTTCAGGCACGAAGG	AGGCAGTCTGTCCTG	AACCTGATGACACAC	TCAGTTAACCAAGGT
761	L Q A R R	R Q S V L	N L M T H	S V N Q G
2341	CAGAACATTCACCGA	AAGACAACAGCATCC	ACACGAAAAGTGTCA	CTGGCCCCTCAGGCA
781	Q N I H R	K T T A S	T R K V S	L A P Q A
2401	AACTTGACTGAACTG	GATATATATTCAAGA	AGGTTATCTCAAGAA	ACTGGCTTGGAAATA
801	N L T E L	D I Y S R	R L S Q E	T G L E I
2461	AGTGAAGAAATTAAC	GAAGAAGACTTAAAG	GAGTGCTTTTTTGAT	GATATGGAGAGCATA
821	S E E I N	E E D L K	E C F F D	D M E S I
2521	CCAGCAGTGACTACA	TGGAACACATACCTT	CGATATATTACTGTC	CACAAGAGCTTAATT
841	P A V T T	W N T Y L	R Y I T V	H K S L I
2581	TTTGTGCTAATTTGG	TGCTTAGTAATTTTT	CTGGCAGAGGTGGCT	GCTTCTTTGGTTGTG
861	F V L I W	C L V I F	L A E V A	A S L V V
2641	CTGTGGCTCCTTGGA	AACACTCCTCTTCAA	GACAAAGGGAATAGT	ACTCATAGTAGAAAT
881	L W L L G	N T P L Q	D K G N S	T H S R N
2701	AACAGCTATGCAGTG	ATTATCACCAGCACC	AGTTCGTATTATGTG	TTTTACATTTACGTG
901	N S Y A V	I I T S T	S S Y Y V	F Y I Y V
2761	GGAGTAGCCGACACT	TTGCTTGCTATGGGA	TTCTTCAGAGGTCTA	CCACTGGTGCATACT
921	G V A D T	L L A M G	F F R G L	P L V H T
2821	CTAATCACAGTGTCG	AAAATTTTACACCAC	AAAATGTTACATTCT	GTTCTTCAAGCACCT
941	L I T V S	K I L H H	K M L H S	V L Q A P

2881	ATGTCAACCCTCAAC	ACGTTGAAAGCAGGT	GGGATTCTTAATAGA	TTCTCCAAAGATATA
961	M S T L N	T L K A G	G I L N R	F S K D I
2941	GCAATTTTGGATGAC	CTTCTGCCTCTTACC	ATATTTGACTTCATC	CAGTTGTTATTAATT
981	A I L D D	L L P L T	I F D F I	Q L L L I
3001	GTGATTGGAGCTATA	GCAGTTGTCGCAGTT	TTACAACCCTACATC	TTTGTTGCAACAGTG
1001	V I G A I	A V V A V	L Q P Y I	F V A T V
3061	CCAGTGATAGTGGCT	TTTATTATGTTGAGA	GCATATTTCCTCCAA	ACCTCACAGCAACTC
1021	P V I V A	F I M L R	A Y F L Q	T S Q Q L
3121	AAACAACTGGAATCT	GAAGGCAGGAGTCCA	ATTTTCACTCATCTT	GTTACAAGCTTAAAA
1041	K Q L E S	E G R S P	I F T H L	V T S L K
3181	GGACTATGGACACTT	CGTGCCTTCGGACGG	CAGCCTTACTTTGAA	ACTCTGTTCCACAAA
1061	G L W T L	R A F G R	Q P Y F E	T L F H K
3241	GCTCTGAATTTACAT	ACTGCCAACTGGTTC	TTGTACCTGTCAACA	CTGCGCTGGTTCCAA
1081	A L N L H	T A N W F	L Y L S T	L R W F Q
3301	ATGAGAATAGAAATG	ATTTTTGTCATCTTC	TTCATTGCTGTTACC	TTCATTTCCATTTTA
1101	M R I E M	I F V I F	F I A V T	F I S I L
3361	ACAACAGGAGAAGGA	GAAGGAAGAGTTGGT	ATTATCCTGACTTTA	GCCATGAATATCATG
1121	T T G E G	E G R V G	I I L T L	A M N I M
3421	AGTACATTGCAGTGG	GCTGTAAACTCCAGC	ATAGATGTGGATAGC	TTGATGCGATCTGTG
1141	S T L Q W	A V N S S	I D V D S	L M R S V
3481	AGCCGAGTCTTTAAG	TTCATTGACATGCCA	ACAGAAGGTAAACCT	ACCAAGTCAACCAAA
1161	S R V F K	F I D M P	T E G K P	T K S T K
3541	CCATACAAGAATGGC	CAACTCTCGAAAGTT	ATGATTATTGAGAAT	TCACACGTGAAGAAA
1181	P Y K N G	Q L S K V	M I I E N	S H V K K
3601	GATGACATCTGGCCC	TCAGGGGGCCAAATG	ACTGTCAAAGATCTC	ACAGCAAAATACACA
1201	D D I W P	S G G Q M	T V K D L	T A K Y T
3661	GAAGGTGGAAATGCC	ATATTAGAGAACATT	TCCTTCTCAATAAGT	CCTGGCCAGAGGGTG
1221	E G G N A	I L E N I	S F S I S	P G Q R V
3721	GGCCTCTTGGGAAGA	ACTGGATCAGGGAAG	AGTACTTTGTTATCA	GCTTTTTTGAGACTA
1241	G L L G R	T G S G K	S T L L S	A F L R L
3781	CTGAACACTGAAGGA	GAAATCCAGATCGAT	GGTGTGTCTTGGGAT	TCAATAACTTTGCAA
1261	L N T E G	E I Q I D	G V S W D	S I T L Q

3841	CAGTGGAGGAAAGCC	TTTGGAGTGATACCA	CAGAAAGTATTTATT	TTTTCTGGAACATTT
1281	Q W R K A	F G V I P	Q K V F I	F S G T F
3901	AGAAAAAACTTGGAT	CCCTATGAACAGTGG	AGTGATCAAGAAATA	TGGAAAGTTGCAGAT
1301	R K N L D	P Y E Q W	S D Q E I	W K V A D
3961	GAGGTTGGGCTCAGA	TCTGTGATAGAACAG	TTTCCTGGGAAGCTT	GACTTTGTCCTTGTG
1321	E V G L R	S V I E Q	F P G K L	D F V L V
4021	GATGGGGGCTGTGTC	CTAAGCCATGGCCAC	AAGCAGTTGATGTGC	TTGGCTAGATCTGTT
1341	D G G C V	L S H G H	K Q L M C	L A R S V
4081	CTCAGTAAGGCGAAG	ATCTTGCTGCTTGAT	GAACCCAGTGCTCAT	TTGGATCCAGTAACA
1361	L S K A K	I L L L D	E P S A H	L D P V T
4141	TACCAAATAATTAGA	AGAACTCTAAAACAA	GCATTTGCTGATTGC	ACAGTAATTCTCTGT
1381	Y Q I I R	R T L K Q	A F A D C	T V I L C
4201	GAACACAGGATAGAA	GCAATGCTGGAATGC	CAACAATTTTTGGTC	ATAGAAGAGAACAAA
1401	E H R I E	A M L E C	Q Q F L V	I E E N K
4261	GTGCGGCAGTACGAT	TCCATCCAGAAACTG	CTGAACGAGAGGAGC	CTCTTCCGGCAAGCC
1421	V R Q Y D	S I Q K L	L N E R S	L F R Q A
4321	ATCAGCCCCTCCGAC	AGGGTGAAGCTCTTT	CCCCACCGGAACTCA	AGCAAGTGCAAGTCT
1441	I S P S D	R V K L F	P H R N S	S K C K S
4381	AAGCCCCAGATTGCT	GCTCTGAAAGAGGAG	ACAGAAGAAGAGGTG	CAAGATACAAGGCTT
1461	K P Q I A	A L K E E	T E E E V	Q D T R L
4441	TAG			
	*			
